# PCV‐VG combined individualized PEEP determination in one‐lung ventilated patients with PEEP step change direction: A randomized controlled trial

**DOI:** 10.1111/crj.13696

**Published:** 2023-09-18

**Authors:** Guowei Li, Saixian Ma, Qian Shu, Zhuhong Fang, Zhiwen Yan, Bo Si

**Affiliations:** ^1^ Department of Anesthesiology Affiliated Wuxi Fifth Hospital of Jiangnan University (Infectious Diseases Hospital of Wuxi) Wuxi Jiangsu China

**Keywords:** one‐lung ventilation, positive end‐expiratory pressure, postoperative pulmonary complications, pressure‐controlled volume guaranteed ventilation

## Abstract

**Introduction:**

The efficacy of pressure‐controlled volume‐guaranteed ventilation (PCV‐VG) combined with a gradient‐directional change in positive end‐expiratory pressure (PEEP) during one‐lung ventilation (OLV) in patients who underwent thoracoscopic surgery was investigated.

**Methods:**

Ninety patients were randomly divided into the PC (PCV‐VG + 5 cm H_2_O fixed PEEP), PI (PCV‐VG + incremental PEEP titration), and PD (PCV‐VG + decremental PEEP titration) groups. Hemodynamic (heart rate [HR] and mean arterial pressure [MAP]), respiratory mechanics (P_peak_, P_mean,_ and Cdyn), and arterial blood gas (pH, PaCO_2_, PaO_2_, and PaO_2_/FiO_2_) indices were evaluated at T_1_ (10 min of two‐lung ventilation [TLV]), T_2_ (10 min of OLV), and T_3_ (10 min of recovery, TLV). Enzyme‐linked immunosorbent assay was performed to detect neutrophil elastase (NE), clara cell secretory protein (CC16), and interleukin‐8 (IL‐8) levels at T1 and T3.

**Results:**

At T_2_ and T_3_, P_peak_ was lower in the PI and PD groups than in the PC group, while P_mean_ and Cdyn were higher than in the PC group. P_peak_ in the PD group was lower than that in the PI group; however, P_mean_ was higher at T_2_ and T_3_ (*P* < 0.05). At T_2_, PaO_2_ and PaO_2_/FiO_2_ were higher, but PaO_2_/FiO_2_ and V_D_/V_T_ were lower in the PD and PI groups than in the PC group (*P* < 0.05). NE, CC16, IL‐6, and IL‐8 levels were elevated in all three groups at T_3_, but the PI and PD groups had lower levels than the PC group (*P* < 0.05). The incidences of postoperative pulmonary complications (PPCs) and surgical intensive care unit hospitalizations in the PD and PI groups were much lower.

**Conclusion:**

Gradient‐directed altered PEEP titration could improve respiratory mechanics, arterial blood gases, and inflammatory responses and reduce the incidence of PPCs in patients undergoing thoracoscopic surgery.

## Graphical abstract

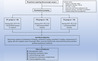

**Abbreviations**
A‐aO_2_
: alveolar‐arterial oxygen partial pressure difference
ARM: alveolar recruitment maneuvers
Cdyn: dynamic compliance
LPV: lung protective ventilation
OI: oxygenation index
OLV: one‐lung ventilation
PaCO_2_
: arterial partial pressure of carbon dioxide
PEEP: Positive end‐expiratory pressure
P_mean_
: airway mean pressure
PPCs: postoperative pulmonary complications
P_peak_
: airway peak pressure
Qs/Qt: intrapulmonary shunt
RI: respiratory index
TLV: two‐lung ventilation
VCV: volume‐controlled ventilation
V_D_/V_T_
: dead space fraction
VT: volume tidal

## 1 INTRODUCTION

Video‐assisted thoracoscopic surgery (VATS) is widely used because of small incisions, low bleeding, and quick patient recovery.^1^ VATs require one‐lung ventilation (OLV) to provide a clear and convenient surgical view and sufficient surgical space to minimize compression or strain on cardiopulmonary tissues during surgery.^2^ In addition, OLV is performed with a double‐lumen bronchial tube or a bronchial blocker to separate the lung on the operative side, effectively isolating the lungs, ensuring oxygen supply to the ventilated lungs, and avoiding the spread of tumor lesions or cross‐infection between the two lungs.^3^ However, non‐physiological ventilation often results in hypoxemia due to increased intrapulmonary shunts and an imbalance in the ventilation‐blood flow ratio.^4^ Furthermore, the incidence of postoperative pulmonary complications (PPCs) associated with OLV is as high as 20%,^5^ seriously affecting the clinical prognosis of patients.

Pressure‐controlled volume‐guaranteed ventilation (PCV‐VG) is a novel ventilation type, combining the advantages of both volume‐controlled ventilation (VCV) and pressure‐controlled ventilation (PCV).^6^ PCV‐VG automatically adjusts airway pressure according to the mechanical parameters of each breath, which is an important part of the protective ventilation strategy.^7^ Numerous studies have shown that positive end‐expiratory pressure (PEEP) increases lung compliance, prevents alveolar collapse, and improves oxygen diffusion, thereby improving pulmonary dynamics and gas exchanges.^8^ However, it is unreasonable to use fixed PEEP for all patients. The optimal PEEP is determined by “PEEP titration” according to the patient's specific situation, which stabilizes lung function, minimizes lung injury, and achieves lung protection.^9^ Optimal PEEP titration determined by lung dynamic compliance (Cdyn) is widely used in clinic practice and has been shown to reduce PPCs in patients undergoing abdominal surgery.^7^ Different PEEP titration methods (incremental or decremental) may produce different results with individual PEEP settings; however, the specific differences in their effectiveness are unclear.

This study was conducted to explore the effects of PCV‐VG combined with direction‐changing PEEP on individualized PEEP determination and lung injury in patients with OLV. The results provide a new perspective on optimizing perioperative anesthesia techniques, reducing intraoperative lung injury, decreasing postoperative complications, and promoting early patient recovery.

## 2 MATERIALS AND METHODS

### 2.1 Research participants

The protocol was approved by the Clinical Trial Ethics Committee of the Affiliated Wuxi Fifth Hospital of Jiangnan University (Infectious Diseases Hospital of Wuxi) (No.2023–003‐1), and written informed consent was obtained from patients or their families. All procedures are in accordance with the Declaration of Helsinki.

Patients undergoing elective thoracoscopic pulmonary surgery from April 2022 to January 2023 were selected based on the following criteria: (1) age ≥ 18 years; (2) body mass index (BMI) < 35 kg/m^2^; (3) American Society of Anesthesiologists (ASA) classification I‐III; and (4) no preoperative blood transfusion. Patient exclusion criteria were as follows: (1) previous history of thoracic surgery; (2) patients with intracorporeal pacemakers; (3) preoperative diagnosis of pneumothorax or pulmonary maculopathy; (4) diagnosis of pulmonary infection, abnormal pulmonary function (FEV1 < 50%), or a history of systemic infection (WBC > 10 × 10^9^ and temperature > 37°C) within the past 1 month; and (5) patients with contraindications to PEEP use, such as high intracranial pressure, hypovolemic shock, and right heart failure. Enrolled patients with any of the following criteria were excluded: (1) surgical approach changed to thoracotomy; (2) intraoperative bleeding ≥ 500 ml; (3) OLV time < 45 min; (4) return to the ward with an endotracheal tube; and (5) peak airway pressure > 40 cm H_2_O on mechanical ventilation during PEEP. According to the above criteria, 90 patients were included in this preliminary study, depending on our ability to recruit patients (a power size calculation revealed a minimum sample size of n = 30 for a power of more than 80% to detect a correlation at a confidence interval of 0.95 and α level of 0.05), retain patients for the duration of the study, and perform intervention and follow‐up. Demographic characteristics, including gender, age, BMI, smoking, alcohol use, and clinical information, are shown in Table 1.

**TABLE 1:** Comparison of the baseline data of subjects.

Parameters	PD (n = 30)	PI (n = 30)	PC (n = 30)	*P* value
Age (year)	55.70 ± 11.16	55.23 ± 11.63	54.11 ± 16.44	0.879
Gender (female/male)	13/17	15/15	12/18	0.730
BMI (kg/m^2^)	22.51 (21.28, 25.83)	22.76 (21.45, 23.90)	23.45 (22.06, 25.05)	0.247
PBW (kg)	57.88 (52.67, 67.15)	65.11 (61.48, 68.97)	64.22 (54.51, 68.76)	0.149
Smoking, n (%)	18 (60.00)	17 (56.67)	15 (50)	0.730
Drinking, n (%)	17 (56.67)	13 (43.33)	17 (56.67)	0.490
ASA (I/II/III)	0/14/16	0/15/15	0/14/16	0.956
Type of surgery, n (%)				
Lobectomy	20 (66.67)	17 (56.67)	14 (46.67)	0.295
Wedge resection	10 (33.33)	13 (43.33)	16 (53.33)	
Volume of fluids (mL)	986.00 ± 317.65	988.00 ± 328.78	984.00 ± 308.36	0.999
Blood loss (mL)	60.00 (50.00, 70.00)	60.00 (50.00, 62.50)	60.00 (50.00, 70.00)	0.312
Urine output (mL)	380.00 (297.50, 485.00)	430.00 (250.00, 502.50)	470.00 (305.00, 520.00)	0.516
Duration of anesthesia (min)	151.60 ± 41.76	147.60 ± 48.22	136.30 ± 45.40	0.401
Duration of operation (min)	134.03 ± 39.57	123.03 ± 40.35	116.73 ± 36.76	0.225
Double lumen tube				
Left/right	26/4	27/3	27/3	0.894
Duration of OLV (min)	130.00 (70.00, 142.50)	115.00 (87.50, 152.50)	125.00 (82.50, 150.00)	0.997

*Note*: Date are presented as mean ± Standard deviation (SD), numbers or median (interquartile range). BMI, body mass index; PBW, predicted body weight, calculated as follows: for women (45.5 + 0.91) × (height in cm‐152.4), for men (50.0 + 0.91) × (height in cm – 152.4); ASA, American Society of Anesthesiologist; OLV: one‐lung ventilation. Two‐way repeated ANOVA followed by post hoc comparison using Fisher LSD Method.

### 2.2 Grouping and anesthetic protocols

Eligible patients were randomly assigned to three study groups (in the ratio of 1:1:1) with a random number generated by using the Statistical Package for Social Sciences software version 22.0 for Windows (IBM SPSS Statistics, 22), based on their registration number in order of referral. The three groups were (i) PC group: as a control group, the patients treated with PCV‐VG and received a conventional lung protective ventilation (LPV) strategy during OLV (maintain 5 cm H_2_O fixed PEEP after alveolar recruitment maneuvers (ARM); (ii) PI group: patients who received PCV‐VG during OLV combined with an OLV ventilation strategy (applying incremental titration of optimal PEEP after ARM); (iii) PD group: patients who received PCV‐VG in combination with a conventional LPV strategy during OLV (applying decremental titration of optimal PEEP after ARM).

Anesthesia and surgical protocols were prepared and performed by the same group of anesthesiologists and surgeons. Electrocardiogram (ECG), heart rate (HR), blood pressure, pulse oxygen saturation (SpO_2_), and bispectral index (BIS) of the patients were monitored immediately upon arrival in the operating room. Peripheral venous access was opened, and a radial artery puncture catheter was placed under local anesthesia to monitor intraoperative arterial pressure and harvest samples for blood gas analysis (Avance CS2 pro; GE Healthcare, Piscataway, NJ, USA). Anesthesia was administered by intravenous injection of 0.05 mg/kg midazolam, 0.5 μg/kg sufentanil, 0.3 mg/kg etomidate, and 0.2 mg/kg cisatracurium. An endotracheal tube was inserted after complete muscle relaxation, and the position and mechanical ventilation were established.

### 2.3 Mechanical ventilation settings

The ventilation parameters were the same in all three groups except for PEEP. First, the V_T_ (tidal volume) was calculated based on the predicted body weight (PBW) according to the following equations: PBW = 50.0 + 0.91 × [height (cm) ‐ 152.4] for males and PBW = 45.5 + 0.91 × [height (cm) ‐ 152.4] for females. The fraction of inspired oxygen (FiO_2_) for two‐lung ventilation (TLV) before OLV was 100%, V_T_ was 8 ml/kg PBW, inspiratory/expiratory ratio was 1:2, PEEP was sustained at 5 cm H_2_O, and all ventilation modes were VCV.

OLV was performed in the lateral position, and the parameters were set as V_T_ 5–6 ml/kg, FiO_2_ 80%, inspiratory/expiratory ratio 1:2, and PEEP values were selected according to the study groups.

ARM was performed at the end of OLV with V_T_ of 8 ml/kg, FiO_2_ of 80%, and an inspiratory/expiratory ratio of 1:2. ARM was performed every 45 mins with the same ventilation pattern and PEEP values as in OLV. During ventilation, the respiratory rate was adjusted to maintain the patient's end‐tidal carbon dioxide partial pressure (P_ET_CO_2_) at 35–45 mmHg.

### 2.4 ARM and PEEP titration

ARM and PEEP titrations were performed in the PI and PD groups. The patient was positioned laterally, and OLV was started after determining and adjusting the position of DLET (double lumen endotracheal tube) using a fiberoptic bronchoscope. An ARM with 5–10 cm H_2_O PEEP is a very useful and simple method for preventing atelectasis and promoting arteriolar oxygenation.^10^ ARM was performed on the ventilated side of the lung, and the ventilation mode was changed to PCV. The driving pressure was 20 cm H_2_O, the inspiratory/expiratory ratio was 1:1, and the respiratory rate was 15 breaths/min. PEEP in the PI group started at 0 cm H_2_O and increased by 2 cm H_2_O every 2 min until the Cdyn value reached its maximum value. PEEP in the PD group started at 16 cm H_2_O and decreased by 2 cm H_2_O every 2 min until the Cdyn value reached its maximum, and the optimal PEEP level was maintained until the end of ventilation. The PC group was subjected to the same ARM but without PEEP titration. After the first ARM, the PEEP value was set to 5 cm H_2_O, and the ARM was performed again 2 min later. The PCV was then changed to PCV‐VG, and a PEEP of 5 cm H_2_O was maintained until the end of ventilation. If the hemodynamics were not stable (> 30% decrease in MAP) during the operation, the operation was discontinued, and 5–15 mg ephedrine or 0.05–15 mg phenylephrine was administered, depending on recovery. Resume of the study protocol (Supplementary data S‐1).

### 2.5 Data collection and measurement

Patients' hemodynamic indices, HR and MAP were recorded at T_1_ (10 min after the initiation of TLV), T_2_ (10 min after the initiation of OLV), and T_3_ (10 min after the initiation of TLV recovery). V_T_, PEEP, airway peak pressure (P_peak_), airway mean pressure (P_mean_), and Cdyn of the patients were also recorded. In addition, the arterial blood gas analyzer I‐STAT 300 (Abbott, USA) was used to record blood gas indicators and ventilation efficiency parameters, such as the arterial partial pressure of carbon dioxide (PaCO_2_), oxygenation index (OI), respiratory index (RI), alveolar‐arterial oxygen partial pressure difference (A‐aO2), and dead space fraction (V_D_/V_T_) during T_1_‐T_3_.^11^, ^12^, ^13^ Parameters were calculated using the following equation: V_D_/V_T_ = (PaCO_2_‐P_ET_CO_2_)/PaCO_2_.

A total of 3 ml of radial artery blood samples were collected in anticoagulation tubes at T_1_ and T_3_. After centrifuging at 2000 rpm for 15 min at 4°C, the supernatant was stored at −80°C. Commercial enzyme‐linked immunosorbent assay (ELISA) kits were employed to analyze the levels of neutrophil elastase (NE), clara cell secretory protein (CC16), and interleukin‐8 (IL‐8).

### 2.6 Clinical endpoints

The primary outcome was the occurrence of postoperative pulmonary complications (PPCs) during hospitalization, including pneumonia, pulmonary atelectasis, acute respiratory failure (ARF), acute respiratory distress syndrome (ARDS), and secondary postoperative tracheal intubation. Mortality within 30 days of surgery was also recorded.

The diagnostic criteria for each item of PPCs were as follows:

Pneumonia: new infiltrating shadows on chest X‐ray and the presence of at least two of the following three indicators: (i) fever (>38.5°C) or hypothermia (<35.58°C); (ii) white blood cell count below 4 × 10^9^/L or > 12.4 × 10^9^/L; (iii) purulent sputum for diagnosis.

Pulmonary Atelectasis: (i) New infiltrative lung shadow on chest radiography accompanied by interlobular fissures shifting towards the affected side. (ii) Management includes bronchoscopy, chest physiotherapy, or mechanical ventilation.

ARF: Dyspnea at rest; respiratory rate > 25 breaths/min, PaO_2_/FiO_2_ ratio < 200, requiring non‐invasive or invasive ventilation (implemented due to hypercapnic acidosis with the partial pressure of carbon dioxide [PaCO2] ≥ 45 mmHg and pH ≤ 7.35).^14^

ARDS was diagnosed according to Berlin's definition: (i) ARF unexplained by cardiac failure or fluid overload; (ii) refractory hypoxemia with PaO_2_/FIO_2_ ≤ 200 mmHg on mechanical ventilation; (iii) bilateral opacities on chest imaging; and (iv) presentation within 1 week of a known clinical impairment or worsening of respiratory symptoms.^14^

Secondary outcomes were the length of stay in the surgical intensive care unit (SICU) and the duration of hospital stay after surgery. Length of stay in the SICU was defined as the physical time spent in the ICU, and the duration of hospital stay was defined as the time from the end of surgery until discharge from the hospital.

### 2.7 Statistical analysis

Statistical analyses were performed using SPSS version 22.0. Statistics of continuous variables conforming to a normal distribution (evaluated by skewness and kurtosis) were described as mean ± standard deviation. One‐way analysis of variance (ANOVA) was used for between‐group comparisons, and the least significant difference‐t method was used for two‐way comparisons. For continuous variables with skewed distributions, statistics were expressed as median and interquartile spacing, and the non‐parametric Kruskal‐Wallis H‐test was employed for two‐way comparisons between groups. Count data were expressed as frequency (rate) and compared using the χ^2^ test. *P* < 0.05 was considered statistically significant.

## 3 RESULTS

### 3.1 The demographic and intraoperative clinical information of patients

**FIGURE 1:**
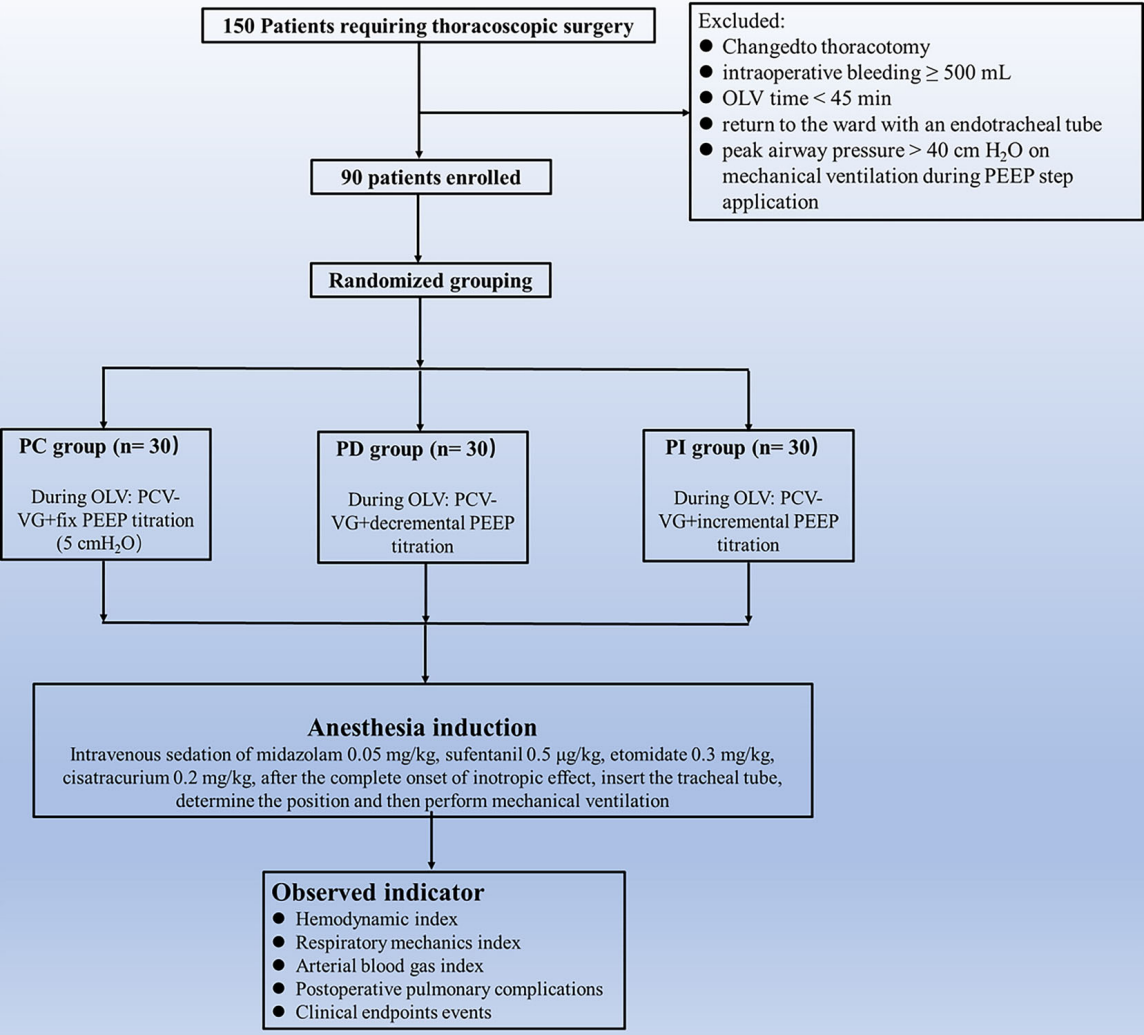
The study flow diagram.

Ninety participants were included in this study (Figure 1). As shown in Table 1, demographic characteristics such as age, gender, BMI, and PBW among the participants in three groups (*P* > 0.05). The operative characteristics, such as the type of surgery, volume of fluids, duration of anesthesia, duration of operation, and duration of OLV were also not significantly different (*P* > 0.05). The results showed that the baseline clinical characteristics of the three groups of patients were comparable preoperatively.

The optimal PEEP obtained using PEEP decremental titration based on Cdyn was 8.7 ± 2.2 cm H_2_O, and the incremental PEEP titration was 8.2 ± 1.3 cm H_2_O, which was significantly different from the fixed 5 cm H_2_O.

### 3.2 Clinical endpoint event

There was no significant difference in pneumonia, atelectasis, ARF, or ARDS among the three groups (*P* > 0.05, Table 2). However, the incidence of total PPCs was significantly lower in the PI and PD groups than in the PC group (*P* < 0.05, Table 2). In addition, the SICU residence times of the PI and PD groups were significantly shorter than those of the PC group (*P* < 0.05, Table 2).

**TABLE 2:** Postoperative pulmonary complications and clinical endpoints events.

Parameters	PD (n = 30)	PI (n = 30)	PC (n = 30)	*P* value
Pneumonia, n (%)	2 (6.67)	3 (10)	5 (16.67)	0.455
Atelectasis, n (%)	2 (6.67)	1 (3.33)	4 (13.33)	0.338
ARF, n (%)	0 (0.00)	1 (3.33)	2 (6.67)	0.355
ARDS, n (%)	0 (0.00)	0 (0.00)	1 (3.33)	0.364
Total PPCs, n (%)	4 (13.33)	5 (16.67)	12 (40.00)	0.044
The duration of SICU stays (hours)	31.00 (28.50, 32.25)*	33.00 (30.00, 34.00)*	39.00 (34.00, 41.00)	< 0.000
The duration of hospital stays after surgery (days)	6.00 (5.00, 7.00)	6.00 (5.00, 7.00)	7.00 (5.00, 8.00)	0.167

*Note*: Date are presented as n (%) or median (interquartile range). ARF, Acute respiratory failure; ARDS, Acute respiratory distress syndrome. Two‐way repeated ANOVA followed by post hoc comparison using Fisher LSD Method.

* *P* < 0.05, compared with PC group.

### 3.3 Hemodynamic indices of the three groups

As shown in Table 3, hemodynamic indices were subsequently analyzed. There was no significant difference in HR at T_1_, T_2_, and T_3_ among the three groups (*P* > 0.05). In addition, the same results were observed for MAP, which was not statistically significant at T_1_, T_2_, or T_3_ (*P* > 0.05). Thus, changes in the direction of PEEP had a slight effect on the hemodynamics of the OLA in the patients, but the difference was insignificant.

**TABLE 3:** Comparison of the hemodynamic index changes between the three groups.

Parameters	PD (n = 30)	PI (n = 30)	PC (n = 30)	*P* value
HR				
T_1_	78.22 (71.75, 82.79)	79.15 (72.28, 81.77)	74.98 (70.78, 80.23)	0.300
T_2_	73.04 ± 6.88	75.12 ± 4.89	74.44 ± 7.82	0.469
T_3_	75.89 ± 8.67	77.61 ± 7.08	74.96 ± 4.93	0.342
MAP				
T_1_	91.78 ± 9.49	94.11 ± 7.74	94.42 ± 8.08	0.421
T_2_	90.62 ± 7.06	72.96 ± 9.94	90.75 ± 7.74	0.477
T_3_	93.77 ± 7.62	94.54 ± 7.60	91.11 ± 8.45	0.217

*Note*: Date are presented as mean ± Standard deviation (SD) or median (interquartile range). HR: heart rate; MAP, mean arterial pressure. Two‐way repeated ANOVA followed by post hoc comparison using Fisher LSD Method.

### 3.4 Ventilation Analysis

There were no significant differences in the respiratory indices, such as P_peak_, P_mean_, and Cdyn, among the three groups at T_1_ (*P* > 0.05). At T_2_, P_peak_ was lower in the PI and PD groups than in the PC group, while P_mean_ and Cdyn were higher (*P* < 0.05) Additionally, P_peak_ was lower in the PD group than that in the PI group; however, P_mean_ was elevated, while Cdyn was not significantly different between the PD and PC groups. Furthermore, at T_3_, decreased P_peak_ and increased P_mean_ and Cdyn were observed in both the PI and PD groups relative to the PC group. Although the differences in P_peak_ and P_mean_ between the PI and PD groups were significant, the difference in Cdyn was not (Table 4).

**TABLE 4:** Comparison of respiratory mechanics indices among the three groups of patients.

Parameters	PD (n = 30)	PI (n = 30)	PC (n = 30)	*P* value
P_peak_ (cmH_2_O)				
T_1_	14.00 ± 3.14	13.83 ± 3.35	14.00 ± 3.72	0.976
T_2_	19.00 (19.00, 20.00)*, ^#^	20.00 (18.75, 20.00)*	22.0 (21.00, 22.25)	< 0.00
T_3_	18.00 (17.00, 19.00)*, ^#^	19.00 (18.00, 19.00)*	20.00 (18.75, 21.00)	< 0.00
P_mean_ (cmH_2_O)				
T_1_	8.00 (7.00, 8.25)	8.00 (7.00, 9.00)	8.00 (7.00, 9.00)	0.481
T_2_	14.00 (13.00, 14.25)*, ^#^	13.00 (12.00, 13.00)*	11.00 (10.00, 12.00)	< 0.00
T_3_	12.00 (11.00, 12.00)*, ^#^	11.00 (10.00, 11.00)*	9.00 (9.00, 10.25)	< 0.00
Cdyn (mL/cmH_2_O)				
T_1_	45.42 (40.51, 48.63)	46.67 (40.55, 50.43)	44.63 (40.98, 46.43)	0.976
T_2_	29.00 (26.00, 31.25)*	28.50 (27.00, 30.25)*	19.50 (19.00, 20.25)	< 0.00
T_3_	47.32 (39.93, 49.83)*, ^#^	44.43 (42.14, 47.08)*	39.69 (37.68, 41.38)	< 0.00

*Note*: Date are presented as mean ± Standard deviation (SD), numbers or median (interquartile range); P_peak_, peak airway pressure; P_mean_, mean airway pressure; Cdyn, dynamic compliance. Two‐way repeated ANOVA followed by post hoc comparison using Fisher LSD Method.

* *P* < 0.05 vs. PC group.

^#^ *P* < 0.05 vs. PI group.

### 3.5 Arterial blood gas variation analysis at different time points

As shown in Table 5, the median PaO_2_ was significantly higher at T_2_ in the PI and PD groups than that in the PC group (*P* < 0.05). In addition, PaO_2_/FiO_2_ was higher in the PI and PD groups than that in the PC group (*P* < 0.05).

**TABLE 5:** Changes of arterial blood gas at different time points for the patients among the three groups.

Parameters	PD (n = 30)	PI (n = 30)	PC (n = 30)	*P* value
PH				
T_1_	7.39 (7.37, 7.42)	7.39 (7.35, 7.43)	7.38 (7.35, 7.41)	0.187
T_2_	7.38 ± 0.03	7.38 ± 0.04	7.39 ± 0.03	0.951
T_3_	7.37 (7.36, 7.40)	7.38 (7.35, 7.39)	7.38 (7.35, 7.42)	0.609
PaCO2 (mmHg)				
T_1_	42.41 (39.79, 44.22)	42.27 (39.74, 47.23)	42.27 (39.74, 47.22)	0.828
T_2_	44.77 ± 3.91	45.99 ± 5.11	45.99 ± 5.11	0.055
T_3_	45.70 (43.14, 47.60)	43.50 (40.98, 46.02)	43.50 (40.98, 46.02)	0.142
PaO_2_ (mmHg)				
T_1_	299.00 (278.50, 307.00)	296.00 (283.00, 307.75)	296.00 (283.00, 307.75)	0.666
T_2_	280.50 (261.25, 293.00)*	276.50 (262.75, 289.25)*	258.00 (243.25, 280.75)	0.018
T_3_	276.00 (257.75, 286.00)*	273.50 (264.50, 286.50)*	260.50 (253.75, 267.25)	0.005
PaO_2_/FiO_2_				
T_1_	329.16 ± 94.39	340.33 ± 78.58	328.83 ± 71.80	0.827
T_2_	174.47 (142.58, 196.27)*	163.42 (148.63, 186.99)*	133.49 (118.89, 163.70)	0.006
T_3_	328.95 (282.56, 369.63)	321.81 ± 77.85	315.93 ± 78.12	0.725

*Note*: Date are presented as mean ± Standard deviation (SD) or median (interquartile range). PaCO_2_, partial pressure of arterial carbon dioxide; V_D_/V_T_, dead space fraction; PaO_2_/FiO_2_, arterial partial pressure of oxygen/fraction of inspired oxygen. Two‐way repeated ANOVA followed by post hoc comparison using Fisher LSD Method.

* *P* < 0.05 vs. PC group.

### 3.6 The serum concentrations of lung V_T_ biomarkers and inflammatory biomarker concentrations

**FIGURE 2:**
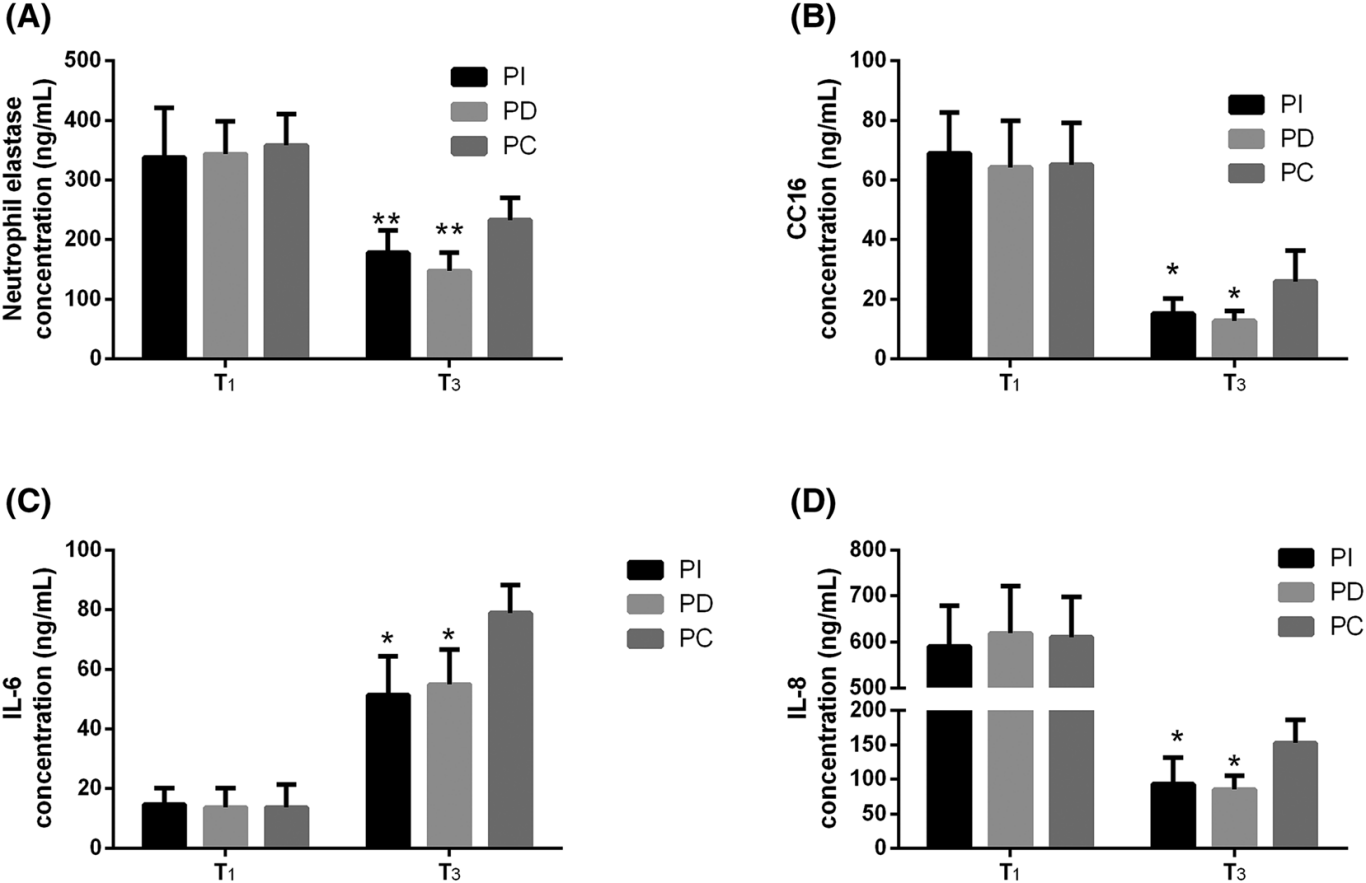
Serum concentration of lung VT biomarkers and inflammatory biomarker concentrations. Perioperative changes in lung VT biomarkers of NE (**A**) and CC16 (**B**). The concentration of inflammatory factor markers IL‐6 (**C**) and IL‐8 (**D**) in three groups of patients during the perioperative period. *** P < 0.05, compared with the PC group.

Concentrations of serum biomarkers associated with pulmonary V_T_, NE, and CC16 were analyzed at T_1_ and T_3_. As illustrated in Figures 2A–B, NE, and CC16 levels were typically higher at T_3_ in all three groups, and the PC group showed higher levels than those in the PI and PD groups (*P* < 0.05). In addition, the serum inflammatory biomarkers IL‐6 and IL‐8 were higher at T_3_, and the PI and PD groups showed lower levels than those in the PC group (*P* < 0.05, Figures 2C–D). PCV‐VG combined with an individualized PEEP ventilation strategy significantly reduced inflammation and lung injury.

## 4 DISCUSSION

This randomized controlled trial found that OLV with increasing or decreasing personalized titration of PEEP in PCV‐VG mode significantly improved pulmonary air exchange and lung mechanics, and reduced serum inflammation, which contributes to an anti‐inflammatory and pulmonary protective effect. In addition, incremental or decremental personalized titration of PEEP in the OLV was effective in suppressing the incidence of postoperative PPCs and reducing the length of stay in the SICU.

VCV, a ventilation model commonly used in clinical practice, ensures stable ventilation but generates high airway pressures, which in turn generates volume injury and leads to uneven gas distribution in the lungs.^15^ PCV reduces airway pressure and decelerates airflow, but may cause lung injury by exerting traction on the alveoli.^16^ However, PCV‐VG combines the advantages of VCV and PCV, which allows the use of decelerated airflow in the volume‐controlled mode and simultaneously ensures V_T_ airflow declaration, reducing airway and alveolar damage from high airway pressures, and protecting the lungs in various fields.^17^ OLV is usually used in thoracic surgery to isolate bilateral lungs and prevent secretions, infection sources, blood, or tumors from one lung from entering the other, thus protecting the healthy lungs. One‐lung anesthesia is required for lung and thoracic surgeries to provide clear vision, shorten the operating time, and reduce unnecessary tissue damage. OLV is an essential form of ventilation for thoracoscopic surgery; however, recent studies have found that improper use of OLV causes lung injury.^18^ Due to shunting on the non‐ventilated side of the lung, some blood is not oxygenated, and pulmonary atelectasis occurs on the ventilated side of the lung, resulting in disproportionate ventilation blood flow, which further impairs arterial oxygenation and promotes hypoxemia.^19^ During anesthesia, when respiratory muscle groups relax and lose tone, the diaphragm moves cephalad, thoracic volume changes, and functional residual air volume and Cydn decrease, eventually causing disproportionate pulmonary ventilation blood flow on the ventilating side.^20^ From the perspective of a pulmonary protective ventilation strategy, appropriate PEEP during OLV is particularly important to prevent mechanical lung injury caused by V_T_ and pulmonary atelectasis.^21^ In addition, high FiO_2_ was used in this study before OLV because of the high hypoxia, and high FiO_2_ is more commonly used in OLV and TLV^22^, ^23^ despite the adverse consequences of high oxygen levels, as reported in previous studies.^24^

Cydn is used clinically as an indicator to assess the efficacy of PEEP pulmonary resuscitation.^25^ Currently, Cydn‐based PEEP titration includes incremental and decremental titration methods.^7^ Previous studies have confirmed that both incremental and decremental titrations reduce intraoperative shunting; however, only decremental titration improves oxygenation and reduces the airway pressure fluctuations required for pulmonary ventilation.^26^ Another recent study suggested that incremental PEEP titration also improves oxygenation^27^ and lung gas distribution uniformity in patients undergoing gynecologic laparoscopy.^28^ Therefore, patients who underwent OLV during thoracoscopic surgery were included in this study and were studied in conjunction with personalized PEEP titration and PCV‐VG. The effectiveness of detecting lung injury using a fixed PEEP titration or incremental or decremental PEEP titrations immediately after ARM was investigated. Additionally, 5 cm H_2_O PEEP was investigated as a fixed PEEP titration based on previous pulmonary protective mechanical ventilation strategies.^29^

In this study, patients were randomized into three groups based on different PEEP titrations. Hemodynamics, respiratory mechanisms, and blood gas indices during the three phases of TLV (T_1_), OLV (T_2_), and TLV (T_3_) were analyzed. No significant differences in hemodynamic indices were found in individualized PEEP titration changes. In contrast, the respiratory indices of patients with incremental and decremental PEEP titrations were lower than those of patients with fixed PEEP titrations. P_mean_ and Cdyn were higher in the T_2_ and T_3_ phases. In our study, respiratory mechanics were improved by personalized PEEP titration and did not affect hemodynamic‐related factors. Consistently, D'Antini and Rauseo conducted two Italian physiology studies in 2018 and found that OLA application titrated PEEP levels improved patient respiratory mechanics but did not affect hemodynamics.^30^, ^31^ We also found a more significant decrease in P_Peak_ and a more significant increase in P_mean_ in patients with decremental PEEP titration than in those with incremental PEEP titration, suggesting that decremental PEEP titration improves respiratory mechanics.

Previous studies have demonstrated that OLV and surgical trauma are accompanied by the release of excess inflammatory cytokines and neutrophil elastase, leading to pulmonary infections and systemic inflammation,^7^, ^32^ which are responsible for postoperative multiorgan dysfunction and other PPCs. Among the many inflammatory factors that have been strongly associated with lung injury and complication, IL‐8 and IL‐6, the former to be catalysts for neutrophils and the latter reported to be a manifestation of the acute phase.^33^ CC16 is an inflammatory mediator that is secreted by Clara cells into the distal lung airway epithelium.^34^ Accumulating evidence has confirmed an association between CC16 and the occurrence of pulmonary atelectasis and hyperinflation in patients undergoing general anesthesia with mechanical ventilation.^7^ As one of the most destructive enzymes, NE is stored in neutrophils under physiological conditions and released during inflammation.^35^ NE breaks down almost the entire extracellular matrix and is an important plasma protein that is released by damaged capillary endothelial cells and alveolar epithelial cells, triggering the onset of acute lung injury.^36^ Large V_T_ leads to hyperinflation of alveoli at the end of inspiration and repeated opening and atrophy of terminal tracheal alveoli, massive infiltration of neutrophils and macrophages, and release of larger amounts of inflammatory mediators, which, in turn, lead to reduced production and secretion of CC16 proteins, lowering lung tissue defenses and accelerating uncontrolled inflammation. NE and CC16 were employed as markers to indirectly evaluate the degree of lung injury in this study, assisted by the analysis of the inflammatory factors IL‐6 and IL‐8. It was also observed that NE, CC16, IL‐6, and IL‐8 levels were elevated in all three groups at T_3_; however, both incremental and decremental PEEP titrations were reduced compared with fixed PEEP.

PPCs are among the most serious problems in the perioperative period and affect patient morbidity as well as lifetime.^22^ Although there is greater controversy as to whether incremental PEEP levels are superior to decremental PEEP titration reported in G. Tusman or F. Peták,^13^, ^37^ PEEP individualization can reduce the incidence, morbidity, and mortality.^38^, ^39^ Herein, no significant differences were found among the three groups of patients for pneumonia, atelectasis, ARF, or ARDS; however, the total PPCs and SICU stay were lower in patients with incremental or decremental PEEP titration.

However, our study had several limitations. First, limited resources constrained us from recruiting sufficient sample sizes to investigate the clinical outcomes of patients with PPCs and other conditions. To ensure data rigor, this study used strict inclusion and exclusion criteria, which may have biased the results. Therefore, the focus of future research should be on conducting multicenter studies with larger sample sizes to explore and elaborate on the various perspectives in depth. In addition, the correlation between cytokine levels and the PEEP, or driving pressure used was missing from this study, which will be addressed in a subsequent study. Surgical procedures and lung atrophy are factors that can affect serum inflammation levels, warranting further investigation in subsequent studies. Additionally, more detailed hemodynamic monitoring, such as emergency fluid pushing, could not be performed during the procedure. Finally, there is a lack of measurement of intrinsic PEEP at zero PEEP.

In conclusion, the combination of PCV‐VG with incremental or decremental PEEP titration ventilation strategies in the OLV showed a favorable effect on intraoperative respiratory mechanics, arterial blood gas indices, and inflammatory response, which reduced the incidence of postoperative PPCs. This study provides a new perspective on ventilation strategies for OLV.

## AUTHOR CONTRIBUTIONS

Guowei Li and Saixian Ma co‐designed this study. Qian Shu and Zhuhong Fang designed the experiment. Guowei Li collected the data and Zhiwen Yan processed the data. Bo Si wrote the manuscript. All authors read and consented to the publication of this study.

## CONFLICT OF INTEREST STATEMENT

The authors have declared no conflict of interest.

## ETHICS STATEMENT

The protocol was approved by the Clinical Trial Ethics Committee of the Affiliated Wuxi Fifth Hospital of Jiangnan University (Infectious Diseases Hospital of Wuxi) (No.2023–003‐1), and written informed consent was obtained from patients or their families. All procedures are in accordance with the Declaration of Helsinki.

## Supporting information


**Data S1** Resume of the study protocol.

**Data S2.** Supporting Information

## Data Availability

The data that support the findings of this study are available from the corresponding author upon reasonable request.
