# Supplementary figures and images for: PCV‐VG combined individualized PEEP determination in one‐lung ventilated patients with PEEP step change direction: A randomized controlled trial
Source: Clin Respir J. 2023 Sep 18;18(1):e13696. doi: 10.1111/crj.13696 (PMC10775886; doi:10.1111/crj.13696)

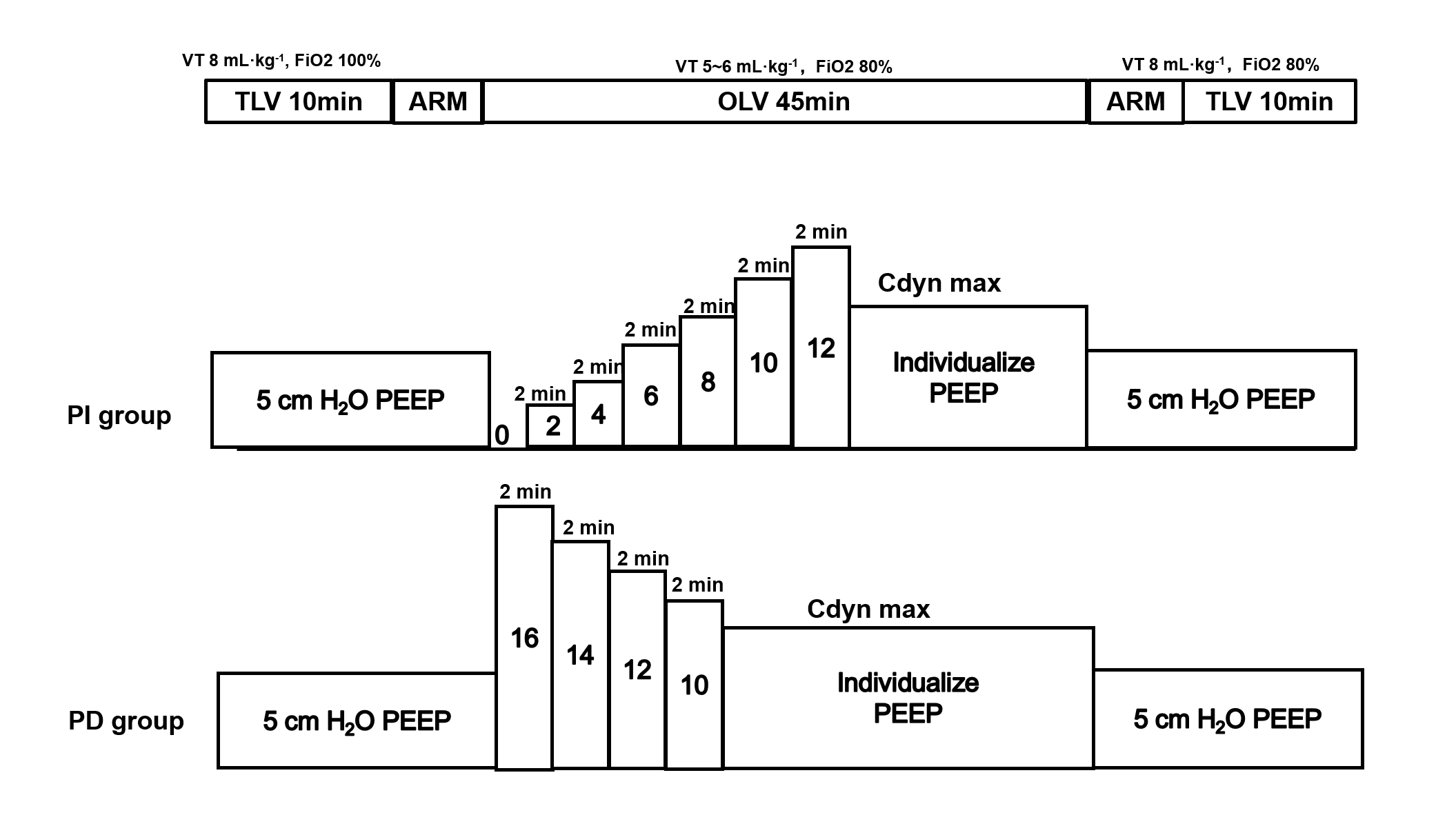

Supplement: Supplementary file 1 — Data S1 Resume of the study protocol. [file CRJ-18-e13696-s001.tif]
