# Supplementary material for: PCV‐VG combined individualized PEEP determination in one‐lung ventilated patients with PEEP step change direction: A randomized controlled trial
Source: Clin Respir J. 2023 Sep 18;18(1):e13696. doi: 10.1111/crj.13696 (PMC10775886; doi:10.1111/crj.13696)
Supplement: Supplementary file 2 — Data S2. Supporting Information [file CRJ-18-e13696-s002.doc]

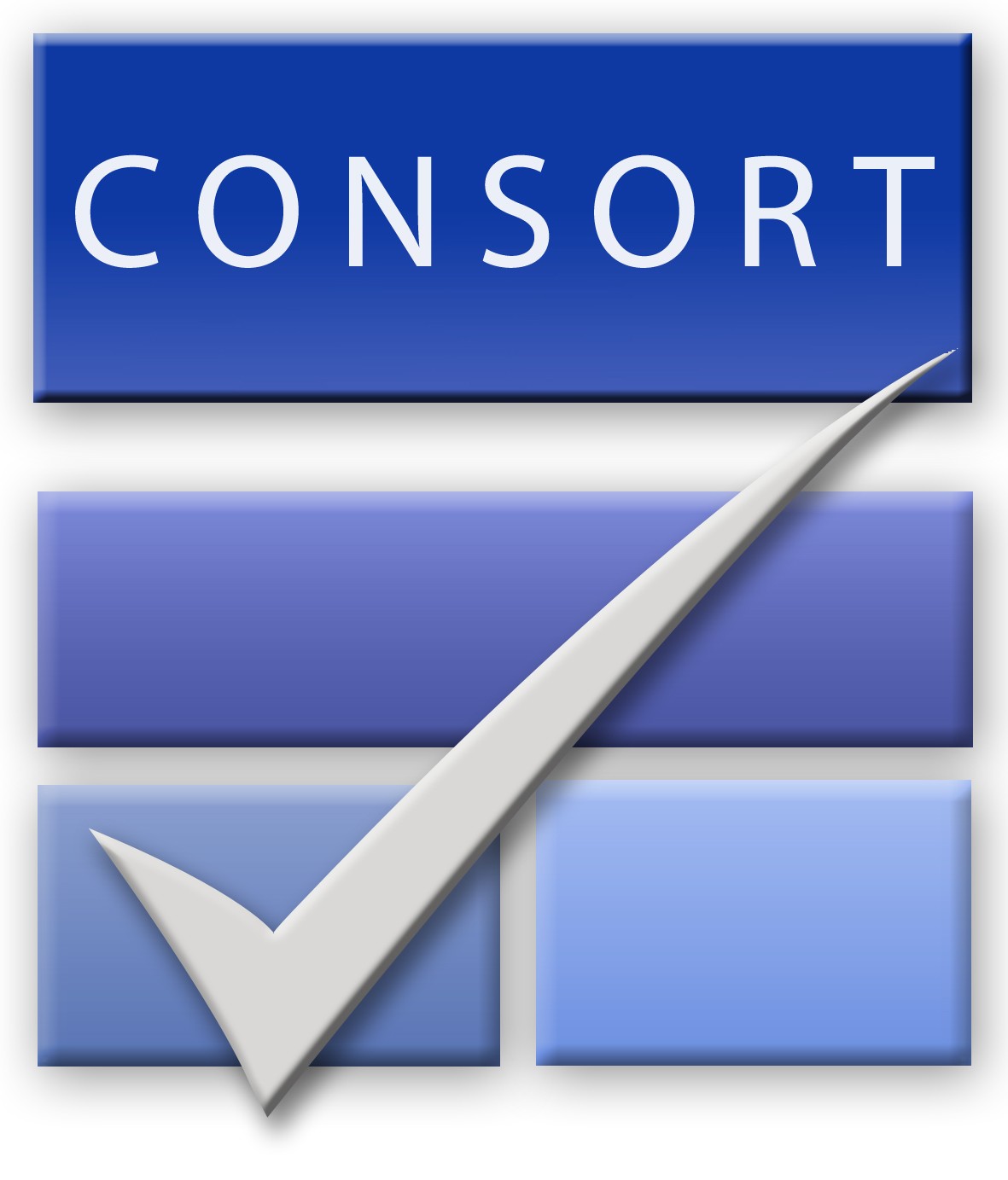
S1. CONSORT Checklist

| Section/Topic | Item No | Checklist item | Reported on page No |
| --- | --- | --- | --- |
| Title and abstract | | | |
|  | 1a | Identification as a randomised trial in the title | Title |
|  | 1b | Structured summary of trial design, methods, results, and conclusions (for specific guidance see CONSORT for abstracts) | Abstract |
| Introduction | | | |
| Background and objectives | 2a | Scientific background and explanation of rationale | Introduction (1 to 2paragraph) |
|  | 2b | Specific objectives or hypotheses | Introduction (3-paragraph) |
| Methods | | | |
| Trial design | 3a | Description of trial design (such as parallel, factorial) including allocation ratio | Methods section: Grouping and anesthetic protocols (1 and 3-paragraph) |
|  | 3b | Important changes to methods after trial commencement (such as eligibility criteria), with reasons | Methods section: Participants in this research (1-paragraph) |
| Participants | 4a | Eligibility criteria for participants | Methods section: Participants in this research (1-paragraph) |
|  | 4b | Settings and locations where the data were collected | Methods section: Data collection and measurement (9-paragraph) |
| Interventions | 5 | The interventions for each group with sufficient details to allow replication, including how and when they were actually administered | Methods section: (3-7-paragraph) |
| Outcomes | 6a | Completely defined pre-specified primary and secondary outcome measures, including how and when they were assessed | Methods section: Clinical endpoint events |
|  | 6b | Any changes to trial outcomes after the trial commenced, with reasons | Methods section: Clinical endpoint events |
| Sample size | 7a | How sample size was determined | Methods section: Participants in this research |
|  | 7b | When applicable, explanation of any interim analyses and stopping guidelines | N/A |
| Randomisation: |  |  |  |
| Sequence generation | 8a | Method used to generate the random allocation sequence | Methods section: Grouping and Anesthetic protocols (3-paragraph) |
|  | 8b | Type of randomisation; details of any restriction (such as blocking and block size) | Methods section: Grouping and Anesthetic protocols (3-paragraph) |
| Allocation concealment mechanism | 9 | Mechanism used to implement the random allocation sequence (such as sequentially numbered containers), describing any steps taken to conceal the sequence until interventions were assigned | Methods section: Grouping and Anesthetic protocols (3-paragraph) |
| Implementation | 10 | Who generated the random allocation sequence, who enrolled participants, and who assigned participants to interventions | Methods section: Grouping and Anesthetic protocols (3-paragraph) |
| Blinding | 11a | If done, who was blinded after assignment to interventions (for example, participants, care providers, those assessing outcomes) and how | Methods section: Grouping and Anesthetic protocols (3-paragraph) |
|  | 11b | If relevant, description of the similarity of interventions | Methods section: “Grouping and Anesthetic protocols” and “Mechanical Ventilation setting” |
| Statistical methods | 12a | Statistical methods used to compare groups for primary and secondary outcomes | Methods section: “Statistical Analysis” |
|  | 12b | Methods for additional analyses, such as subgroup analyses and adjusted analyses | N/A |
| Results | | | |
| Participant flow (a diagram is strongly recommended) | 13a | For each group, the numbers of participants who were randomly assigned, received intended treatment, and were analysed for the primary outcome | Methods section: “Clinical endpoint event” (6-paragraph) |
|  | 13b | For each group, losses and exclusions after randomisation, together with reasons | Graphical abstract |
| Recruitment | 14a | Dates defining the periods of recruitment and follow-up | **Recruitment:** Results_’participants in this research’ (1-paragraph)  **Follow-up:** Methods_’Clinical endpoints event’ |
|  | 14b | Why the trial ended or was stopped | N/A |
| Baseline data | 15 | A table showing baseline demographic and clinical characteristics for each group | Table 1 |
| Numbers analysed | 16 | For each group, number of participants (denominator) included in each analysis and whether the analysis was by original assigned groups | Table 1-5 |
| Outcomes and estimation | 17a | For each primary and secondary outcome, results for each group, and the estimated effect size and its precision (such as 95% confidence interval) | Table 1-5 |
|  | 17b | For binary outcomes, presentation of both absolute and relative effect sizes is recommended | N/A |
| Ancillary analyses | 18 | Results of any other analyses performed, including subgroup analyses and adjusted analyses, distinguishing pre-specified from exploratory | N/A |
| Harms | 19 | All important harms or unintended effects in each group (for specific guidance see CONSORT for harms) | N/A |
| Discussion | | | |
| Limitations | 20 | Trial limitations, addressing sources of potential bias, imprecision, and, if relevant, multiplicity of analyses | Discussion_(7-paragraph) |
| Generalisability | 21 | Generalisability (external validity, applicability) of the trial findings | Discussion_(1-paragraph) |
| Interpretation | 22 | Interpretation consistent with results, balancing benefits and harms, and considering other relevant evidence | Discussion_(1-6 paregraph) |
